# The Non-Obese Diabetic Mouse Strain as a Model to Study CD8^+^ T Cell Function in Relapsing and Progressive Multiple Sclerosis

**DOI:** 10.3389/fimmu.2015.00541

**Published:** 2015-10-22

**Authors:** Prenitha Mercy Ignatius Arokia Doss, Andrée-Pascale Roy, AiLi Wang, Ana Carrizosa Anderson, Manu Rangachari

**Affiliations:** ^1^Department of Neurosciences, Centre de recherche du CHU de Québec – Université Laval (Pavillon CHUL), Québec, QC, Canada; ^2^Ann Romney Center for Neurologic Diseases, Brigham and Women’s Hospital and Harvard Medical School, Boston, MA, USA; ^3^Evergrande Center for Immunologic Diseases, Brigham and Women’s Hospital and Harvard Medical School, Boston, MA, USA; ^4^Department of Molecular Medicine, Faculty of Medicine, Université Laval, Québec, QC, Canada

**Keywords:** multiple sclerosis, relapsing–remitting multiple sclerosis, progressive multiple sclerosis, experimental autoimmune encephalomyelitis, CD8^+^ T cell, CD4^+^ T cell, non-obese diabetic mouse, 1C6

## Abstract

Multiple sclerosis (MS) is a neurodegenerative disease resulting from an autoimmune attack on central nervous system (CNS) myelin. Although CD4^+^ T cell function in MS pathology has been extensively studied, there is also strong evidence that CD8^+^ T lymphocytes play a key role. Intriguingly, CD8^+^ T cells accumulate in great numbers in the CNS in progressive MS, a form of the disease that is refractory to current disease-modifying therapies that target the CD4^+^ T cell response. Here, we discuss the function of CD8^+^ T cells in experimental autoimmune encephalomyelitis (EAE), a mouse model of MS. In particular, we describe EAE in non-obese diabetic (NOD) background mice, which develop a pattern of disease characterized by multiple attacks and remissions followed by a progressively worsening phase. This is highly reminiscent of the pattern of disease observed in nearly half of MS patients. Particular attention is paid to a newly described transgenic mouse strain (1C6) on the NOD background whose CD4^+^ and CD8^+^ T cells are directed against the encephalitogenic peptide MOG_[35–55]_. Use of this model will give us a more complete picture of the role(s) played by distinct T cell subsets in CNS autoimmunity.

## Introduction

Multiple sclerosis (MS) is a complex chronic neurological disease that results from an immune-mediated attack against central nervous system (CNS) myelin. It is characterized by demyelination, axon damage, white matter inflammation, and glial scarring (1). Approximately 2.5 million people in the world are affected by MS (2) and it is therefore important that we better understand its causes, with the goal of developing treatments that can improve disease outcomes. The four known subtypes of MS are relapsing–remitting (RR), secondary progressive (SP), primary progressive, and progressive relapsing. Approximately 85% of MS patients display an RR disease course in which repeated periods of inflammatory response are followed by remission. Nearly 50% of RR patients eventually transition to a chronic SP phase marked by slow and steady increase in disability, and more than half of all MS patients will exhibit some form of progressive disease (3). Although CD4^+^ T helper cells have been thought to be the main players in the pathogenesis of MS, the evidence suggests that disease processes in MS involve other immune cell types that include, among others, CD8^+^ T cells, B cells, macrophages, microglia, and neutrophils (4). Our goal here is to discuss the role of CD8^+^ T cells in the pathology of MS with a particular focus on findings from studies of experimental autoimmune encephalomyelitis (EAE), a mouse model of MS.

## T Cells in MS Pathogenesis

Autoimmunity results from (a) the escape of self tissue antigen-specific T cells from negative selection and deletion in the thymus and (b) the breakdown of peripheral immune tolerance mechanisms, such as inhibitory receptor signaling and regulatory T cell-mediated inhibition. MS specifically results from an autoreactive T cell inflammatory reaction against myelin-producing CNS oligodendrocytes (1). The role of T cells in MS pathogenesis is strongly supported by genetic analysis demonstrating that polymorphisms in the human leukocyte antigen (HLA) gene region (5) and in genes encoding T cell-related signaling molecules and cytokines (6) which are strongly linked to MS susceptibility. Furthermore, immunohistochemical analysis of acute and recent MS lesions reveal extensive perivascular infiltration of T lymphocytes (7).

Myelin-reactive T cells proliferate and differentiate into effector subpopulations in peripheral lymphoid tissues, where they recognize myelin-derived antigenic epitopes presented by specialized antigen-presenting cells. The CD4^+^ IFN-γ-secreting Th1 and IL-17-secreting Th17 subsets have been well described in both MS (8, 9) and EAE (10). However, CD8^+^ T cells can also respond vigorously to myelin antigen and induce neuroinflammatory damage (11). Activated T cells express cell adhesion molecules (e.g., LFA-1, VLA-4, and PSGL-1) and chemokine receptors (e.g., CCR5 and CXCR3) that permit them to cross the blood brain barrier (12). Once in the CNS, they are reactivated by local antigen-presenting cells, such as microglia and dendritic cells (13), which themselves produce proinflammatory cytokines (e.g., IFN-γ, IL-23, TNF-α, and lymphotoxin-α) and chemokines (e.g., RANTES, CXCL10, and IL-8). This attracts other immune effector cells from the circulation (12–14).

It has been more than 20 years since the first effective disease-modifying therapies were introduced for RR–MS. Both IFN-β and glatiramer acetate appear to modulate the function of inflammatory T cells (15, 16). More recently developed drugs, such as natalizumab (17) and rituximab (18), also target lymphocyte function, thus emphasizing the critical role played by dysregulated adaptive immune responses in MS. However, there are no currently available treatments for progressive MS, which has led some to argue that this form of the disease is driven by neurodegenerative rather than inflammatory mechanisms (19). On the other hand, lymphocytes accumulate in the meninges (20–22) and CD3^+^ T cells are detected in normal-appearing white matter of the spinal cord during progressive disease (20). Intriguingly, CD8^+^ T cells are detected in normal-appearing white matter, active lesions, and inactive lesions in progressive MS (23), and can directly induce demyelination (24). Thus, CD8^+^ T cell function may present an attractive target for the treatment of progressive MS.

## CD8^+^ T Cells in MS

A number of current MS drugs, such as interferon-β (15), glatiramer acetate (16), and natalizumab (17), appear to modulate CD4^+^ T cell responses, indicating the relevance of these cells to pathogenesis. However, multiple lines of evidence suggest that CD8^+^ T cells also play a key role in MS pathology. On the genetic level, positivity for the HLA class I allele A3 increases one’s risk of developing MS (25, 26); as the human CD8^+^ T cell repertoire is restricted by class I molecules, this implies that CD8^+^ T cell reactivity to specific myelin antigens can predispose an individual to developing MS. Myelin-specific CD8^+^ T cells show oligoclonal expansion in plaques, cerebrospinal fluid (CSF), and blood of MS patients, and the frequency of CD8^+^ T cells greatly exceeds that of CD4^+^ T cells in acute MS lesions (27). Interestingly, CD8^+^ T cells are detected in NAWM in MS brains, suggesting that they are among the first lymphocytes on the scene in the earliest stages of disease (23). In addition, during MS, CD8^+^ T cells upregulate cell adhesion molecules involved in immune trafficking into the CNS; increased frequency of CCR5^+^ and CXCR3^+^ CD8^+^ T cells in peripheral blood correlates with increased annualized MS lesion load (28) and increased PSGL-1^+^CD8^+^ T cell frequency is observed in active MS (29).

CD8^+^ T cells can execute inflammatory damage in the CNS via two distinct mechanisms: a direct mechanism by which they attack MHC class I-expressing axons, or an indirect one by which they attack oligodendrocytes, thereby exposing axons to further damage (30). Granzyme B-positive CD8^+^ T cells are found in close proximity to demyelinated axons in MS lesions and their cytolytic granules appear to be polarized toward the site of injury (24). CSF levels of granzymes A and B are elevated during active MS (31) and highly differentiated CD8^+^ T cells are enriched in the CSF during early MS (32). Importantly, various cells of the CNS, including neurons, astrocytes, and oligodendrocytes, can be induced to express MHC class I on their surface in the context of an inflammatory response. These cells are, thus, susceptible to CD8^+^ T cell-mediated killing (24). Indeed, abundance of CD8^+^ T cells in MS brain tissue positively correlates with the extent of axonal damage (33). Taken together, these data suggest that CD8^+^ T cells proliferate in response to myelin antigens, and traffic to the CNS, where they can help to initiate and maintain tissue inflammation and damage.

## Animal Models of MS

Experimental autoimmune encephalomyelitis is a murine disease that recapitulates the immunopathogenesis of MS. It can be induced (a) by active immunization with encephalitogenic peptides derived from myelin component proteins, such as myelin basic protein (MBP), proteolipid protein (PLP), or myelin oligodendrocyte glycoprotein (MOG) or (b) by passive (“adoptive”) transfer of myelin-reactive lymphocytes to genetically susceptible recipient mice (34). Classic adoptive transfer approaches entail the isolation and re-stimulation of myelin-specific lymphocytes from actively immunized donor mice, followed by transfer to genetically susceptible recipient mice. As will be described below, EAE can also be induced by the adoptive transfer of transgenic, myelin antigen-reactive, T cells.

Immunization of C57BL/6J (B6) mice with MOG_[35–55]_ is a popular model of EAE induction, in part because of the ready availability of genetically modified strains on the B6 background (34). These mice typically develop an ascending paralysis that is either monophasic or chronically non-remitting (35), which does not reflect the full clinical course of MS. By contrast, immunization of SJL/J mice with PLP_[139–151]_ induces a RR disease pattern characterized by epitope spreading of the immune response to secondary myelin antigens (36). Furthermore, as we will discuss, immunization of non-obese diabetic (NOD) background mice with MOG_[35–55]_ induces a RR → SP disease course characterized by extensive demyelination and axonal loss (37). This disease course is reminiscent of that seen in close to half of MS patients (3).

In recent years, the field has welcomed the introduction of T cell receptor (TcR) transgenic (Tg) mouse strains, such as MBP-reactive Ac1-11 (38) and T/R (39), PLP-reactive 5B6 (40), and MOG-reactive 2D2 (41) and 1C6 (42), in which >90% of an animal’s T cells are directed against a defined myelin epitope, thus providing a readily available source of myelin antigen-specific T cells for adoptive transfer. Although EAE develops spontaneously on the T/R Tg strain (39) and can be observed at a low spontaneous frequency in 2D2 mice, the disease typically requires robust induction of T cell activation, either via the use of adjuvants that stimulate the innate immune system or by the *ex vivo* triggering of TcR and costimulatory receptors on myelin-reactive T cells (10, 43). No single model of EAE can recapitulate the complex clinical presentation of MS in its entirety. However, depending on the immunogen used and the method of induction, one can observe paralysis of the extremities (44), ataxia (10), optic nerve inflammation (45), and weight loss (46).

Although EAE models have yielded invaluable insights into the role of autoreactive CD4^+^ T cells in pathogenesis, they have been less forthcoming with respect to the role of CD8^+^ T cells. This is in part because 21–23mer peptides, such as MOG_[35–55]_ or PLP_[139–151]_, are optimally presented by MHC class II molecules. Nevertheless, Ford et al. demonstrated that adoptive transfer of CD8^+^ T cells from MOG_[35–55]_-immunized B6 mice into severe combined immunodeficient (SCID) recipients resulted in severe EAE, and found that the pathogenic CD8^+^ T cells recognize a minimal core epitope MOG_[37–46]_ in the context of class I H-2D^b^ (47). Notably, CD8^+^ T cells from MOG_[35–55]_-immunized NOD mice elicited reactivity toward a core epitope MOG_[39–47]_ (48).

Early models, involving the transfer of ectopic Ag-specific T CD8^+^ T cells to Tg mice expressing the ectopic Ag in CNS tissue, supported a role for CD8^+^ T cells in CNS inflammation (49). More recently, models with CD8^+^ T cells directed at myelin Ag have been described, such as Tg mice (strain 8.8) that express a MHC class I-restricted TcR specific for MBP_[79–87]_ (46). Infection of these mice with a recombinant vaccinia virus encoding MBP efficiently induced EAE. Intriguingly, wild-type (WT) vaccinia virus induced EAE in 8.8 mice as well. The effect appeared to be due to the expression of endogenous TcR chains, as WT virus did not cause EAE in 8.8 × *Rag1*^−/−^ mice and as CD8^+^ T cells co-expressing 2 distinct β-chains (Vβ8 and Vβ6) were detected in WT virus-infected mice. Their findings suggest a cellular mechanism by which dual antigen-specific CD8^+^ T cells can be initially activated in response to virus and then induce damage to CNS tissue, and thus reveal a potential mechanism by which viral infection can trigger CD8^+^ responses directed against myelin. More recently, a Tg strain (BG1) was described that carries CD8^+^ T cells specific for astrocytic glial fibrillary acidic protein (50). These mice spontaneously develop a RR disease course characterized by lesions in both CNS white and gray matter. Intriguingly, B6 mice adoptively transferred with BG1 CD8^+^ T cells develop atypical EAE and suffer from weight loss upon inoculation with Vac-GFAP, in a manner reminiscent of Vac-MBP-inoculated 8.8 mice (46). However, WT Vac does not induce symptoms in BG1 mice, indicating that this phenotype was not caused by the expression of endogenous TcR β-chains (50). The relevance of CD8^+^ T cells to virally induced CNS autoimmune pathology is further supported by Pirko et al., who infected B6 mice with Theiler’s murine encephalomyelitis virus and found that the presence of T1 “black holes” on MRI – a classic sign of chronic and irreversibly damaged lesions – correlated with the accumulation of CD8^+^ T cells (51). As epitopes 35–55 of MOG are located in its extracellular domain, in the future it would be interesting to examine whether MHC class I-restricted peptides derived from myelin-associated glycoprotein (MAG) or PLP, both of which have extracellular domains (52), could induce CD8^+^ T cell-driven EAE.

## NOD Mice as a Model for Autoimmune Disease

Type 1 diabetes (T1D) is an autoimmune disease initiated by the infiltration of mononuclear cells into the pancreatic islets of Langerhans (insulitis). This is followed by autoreactive T cell-mediated destruction of insulin-producing pancreatic β-cells (53). T1D and MS have been linked epidemiologically. A large-scale Danish study found that T1D patients had a threefold-greater risk of developing MS (54), and a study of Sardinian MS patients found that they were approximately threefold more likely to have T1D than their healthy siblings (55). Indeed, MS and T1D share common etiological factors, such as increased incidence at northern latitudes and among individuals born in springtime (56). Furthermore, MS patients have islet-antigen-reactive T cells in their repertoire, whereas T1D patients have T cells that respond to myelin-derived epitopes (57). Taken together, these findings suggest that MS and T1D may share common pathogenic mechanisms.

The NOD strain has been used as a model for T1D for over three decades (58). NOD mice spontaneously develop T cell responses to the β-cell antigen GAD65, resulting in insulitis and subsequent β-cell destruction (59). NOD mice can also develop self-reactive inflammatory responses to a variety of tissues, and EAE can be induced in these animals upon active immunization with MOG_[35–55]_ (60, 61). More than 20 non-MHC genetic loci (termed *Idd*) have been identified that can contribute to T1D pathogenesis on the NOD strain (62). In an elegant series of experiments, Encinas et al. immunized a series of congenic NOD mice carrying B6-derived *Idd* loci with MOG_[35–55]_ (60). They found that NOD mice carrying B6-origin *Idd3* developed milder EAE than WT NOD mice. In contrast to WT NOD, B6-*Idd3* mice did not develop chronic disease, suggesting that differences in this genetic region were potentially responsible for the development of progressive EAE in NOD mice. Thus, the immunoregulatory factor(s) encoded in the *Idd3* locus have been the subject of intense interest. The gene encoding the T cell growth factor IL-2 is located in *Idd3*, and there is a coding polymorphism between the B6 and NOD *Il2* genes (60). NOD.*Idd3* lymphocytes transcribe twofold more *Il2* than WT NOD mice. This seemingly paradoxical observation is explained by the findings that excess IL-2 in NOD.*Idd3* mice increases the function of IL-2-dependent FoxP3^+^ regulatory T cells, which suppress pancreatic inflammation (63), and that IL-2 responsive macrophages can suppress inflammatory Th17 responses (64). IL-21 is also encoded in the Idd3 locus (65), and NOD.*Idd3* T cells produce less IL-21 than their WT NOD counterparts. This reduction in IL-21 results in defective NOD.*Idd3* Th17 responses due to both T cell-intrinsic and -extrinsic mechanisms (66). Furthermore, IL-21 plays a crucial role in CD8^+^ T cell-driven T1D (67). Thus, the *Idd3* locus can likely restrict tissue inflammation in both the pancreas and CNS through multiple regulatory mechanisms.

## EAE in NOD Mice

Upon immunization with MOG_[35–55]_, NOD mice develop RR–EAE that progresses to a chronic SP disease course characterized by loss of both axons and myelin as measured by magnetic resonance imaging (37). During the initiation phase, inflammatory cells infiltrate the perivascular and arachnoid space, and splenocyte production of IFN-γ, TNF-α, and IL-6 is enhanced. By contrast, IL-17 is upregulated in re-stimulated splenocytes during relapses that follow the initiation phase. These relapses are characterized histologically by mononuclear cell infiltration into the white matter and demyelination (68). However, CNS-resident cells, such as microglia and astrocytes, also participate in the transition from RR to SP phase in NOD–EAE, with reactive gliosis being present from the early stage of the disease (69).

Both CD8^+^ and CD4^+^ T cells appear to play important roles in mediating EAE on the NOD background. NOD mice deficient in key regulators of CD4^+^ T cell function show alterations in their susceptibility to EAE (70, 71). Immunization with MOG_[35–55]_ causes the expansion of an IFN-γ^+^ CD8^+^ T cell population in NOD mice that recognize the core epitope MOG_[39–47]_ (48). Our preliminary data reveal the infiltration of inflammatory effector CD44^hi^ CD8^+^ T cells into the CNS upon active immunization with MOG_[35–55]_. These CD8^+^ T cells display a Tc1 phenotype with the production of proinflammatory cytokines, such as IFN-γ, TNF-α, and IL-2 (unpublished).

## 1C6 TCR Transgenic Mice

**Figure 1:**
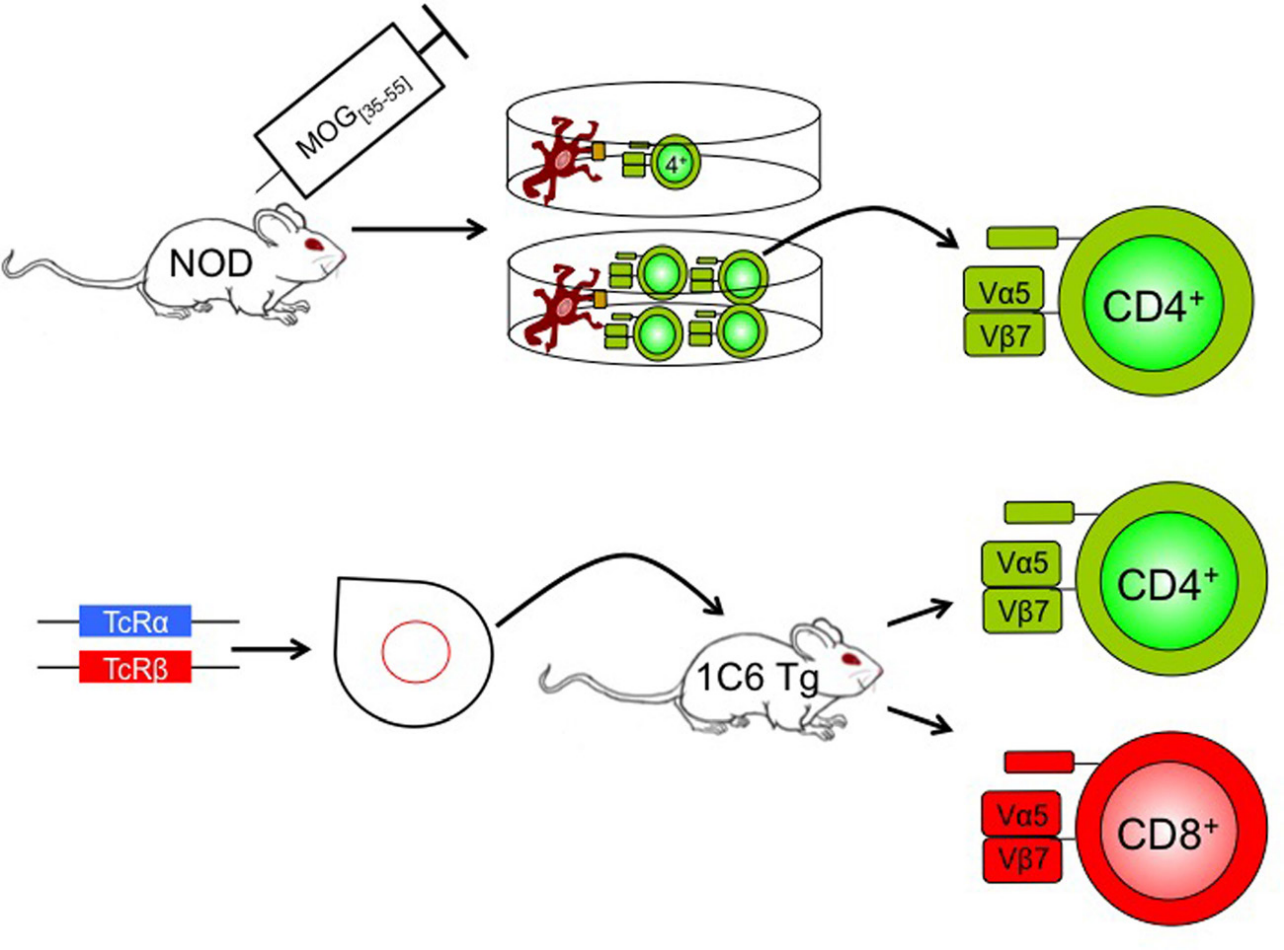
**Generation of 1C6 mice**. A NOD mouse was actively immunized with MOG_[35–55]_. CD4^+^ T cells were isolated from the immunized mouse and were re-stimulated at one T cell per well with antigen-presenting cells plus MOG_[35–55]_. The T cell receptor of an expanding clone was sequenced (Vα5Jα22; Vβ7Dβ2Jβ2.7), cloned, and injected into NOD pronuclei to derive 1C6 transgenic mice. The resulting mice possessed both CD4^+^ and CD8^+^ T cells that were MOG_[35–55]_ reactive. Tg, transgenic; TcR, T cell receptor.

Recently, Anderson et al. generated a novel TcR transgenic mouse model on the NOD background, using the TCRα and TCRβ chains of a MOG_[35–55]_ CD4^+^ clone (42). Surprisingly, the resulting strain (named 1C6) possesses both CD4^+^ and CD8^+^ T cells with specificity for MOG_[35–55]_ (Figure 1), with the proportion of CD4^+^/CD8^+^ T cells in peripheral lymphoid tissues being similar to that seen in WT animals. The 1C6 transgenic strain is thus the first to have both myelin-reactive CD4^+^ and CD8^+^ T cells, allowing us to interrogate the relative contribution of both cell types to EAE. Active immunization of 1C6 mice with MOG_[35–55]_ induces a RR to SP disease as seen in NOD mice. Furthermore, 1C6 mice develop spontaneous EAE and optic neuritis at a frequency of about 2%. Adoptive transfer of 1C6 CD4^+^ T cells, CD8^+^ T cells, or both together, to lymphocyte deficient NOD.Scid mice, followed by immunization with MOG_[35–55]_, results in the development of EAE. Both the incidence and severity of EAE were lower in 1C6 CD8^+^ T cell transfer recipients when compared to mice receiving 1C6 CD4^+^ T cells alone or 1C6 CD4^+^ plus CD8^+^ T cells. It remains to be seen whether immunization with the class I-restricted MOG_[39–47]_ peptide (48) could induce disease of greater incidence and/or severity in NOD.Scid mice reconstituted with 1C6 CD8^+^ T cells given that the 21-mer MOG_[35–55]_ peptide is optimally presented by MHC class II molecules. 1C6 CD8^+^ T cells did have the capacity to induce optic neuritis; furthermore, they produced IFN-γ and granzyme B, and degranulated, in response to MOG_[35–55]_ (42).

**Figure 2:**
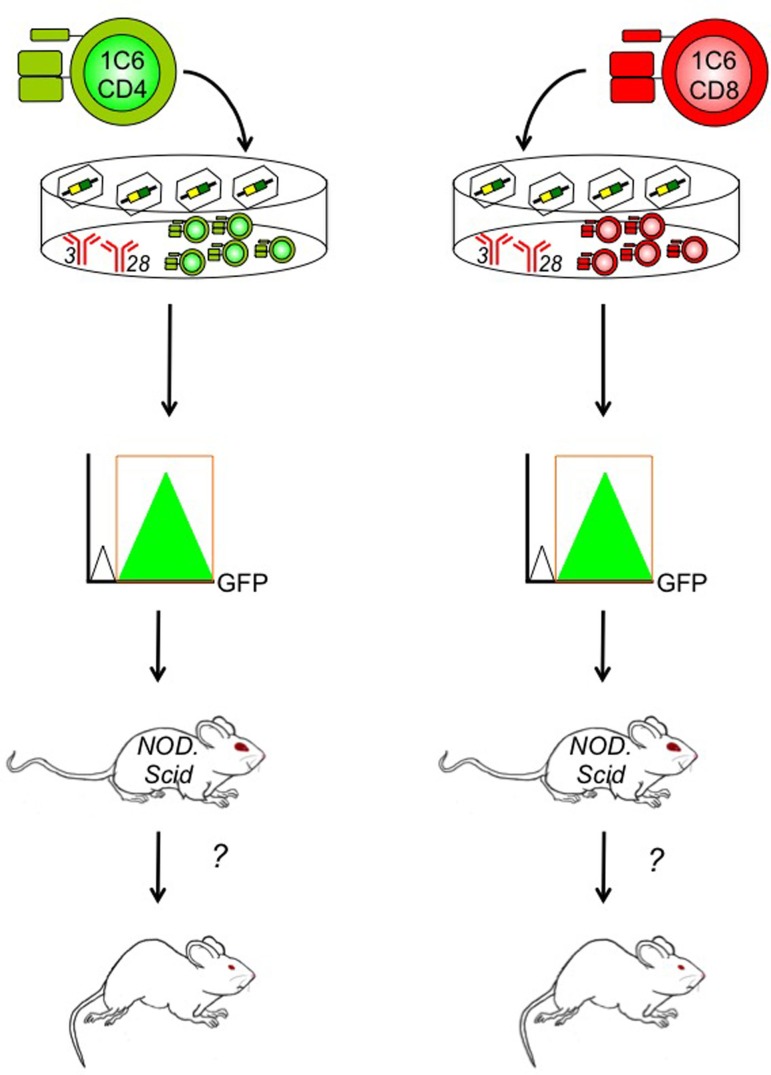
**Determining the contribution of specific genes to CD4^+^ and/or CD8^+^ T cell-driven EAE using the 1C6 model**. CD4^+^ (left) and/or CD8^+^ T cells (right) are isolated from the peripheral lymphoid tissue of 1C6 mice. They are stimulated *ex vivo* using plate-bound anti-CD3 and anti-CD28 monoclonal antibodies that mimic the physiological signals required for T cell proliferation. They are coincidentally transduced with gene-specific retroviruses that can augment or knock down expression of the candidate molecule. Transduced 1C6 T cells are purified using high-speed cell sorting based on expression of retrovirally encoded bicistronic reporters, such as GFP. They are then transferred to NOD.Scid mice that are assessed for the development of EAE. This design can help us isolate the function of candidate T cell regulatory molecules to CD4^+^ T cells, CD8^+^ T cells, or both, in the context of MS-like disease.

Importantly, the 1C6 model will enable us to distinguish between the molecular pathogenesis of CD4^+^ versus CD8^+^ T cell-driven CNS autoimmunity. CD4^+^ and CD8^+^ T cells express many of the same effector molecules; it is therefore difficult to draw conclusions on the relative contribution of the two cell types based on active immunization of whole-animal gene knockouts. Furthermore, T-cell-specific transgenic deleter strains, such as *Lck*-Cre and *CD4*-Cre, target gene expression in both CD4^+^ and CD8^+^ T cells (72, 73). To address this gap in the field, we have developed an EAE induction protocol in which T cells are isolated from 1C6 mice, are stimulated and differentiated *ex vivo* into defined effector subsets (Th1 or Th17 CD4^+^; Tc1 or Tc17 CD8^+^), and are then adoptively transfer to NOD.Scid mice (unpublished data). Using retrovirally mediated gene transduction, we can now manipulate the expression of a target gene in 1C6 CD4^+^ T cells, CD8^+^ T cells, or both concomitantly, to assess in which cell type the molecule of interest exerts its effects (Figure 2). This will allow us to ascertain in which T cell compartment a given immune regulatory molecule exerts its function, in a mouse model that can recapitulate both the relapsing/remitting and progressive phases of MS.

## Conclusion

Several decades’ worth of evidence from animal models have supported the idea that CD4^+^ T cells are the chief drivers of inflammation in MS. However, histopathological and clinical findings from human patients indicate that CD8^+^ T cells are key players as well. Indeed, several popular therapeutic reagents that are believed to subvert CD4^+^ T cell function in MS – namely, interferon-β, natalizumab, and alemtezumab – could also exert effects on CD8^+^ T cells. Furthermore, to date, no effective treatments exist for progressive MS, and there are indications that CD8^+^ T cells play a role in tissue damage during this disease state (23). Thus, targeting autoreactive CD8^+^ T cells could present an attractive mechanism by which to treat progressive MS.

The 1C6 mouse model will allow us to examine this possibility. As these mice possess both CD8^+^ and CD4^+^ myelin-reactive T cells, we can address both the role of CD8^+^ T cells in CNS autoimmunity and the pathophysiology of progressive MS. In particular, genetic or pharmacological manipulation of 1C6 T cells permits us to interrogate the function of candidate genes or signaling pathways in both CD4^+^ and CD8^+^ T cell-driven EAE, simultaneously or separately, in the same animal. Such an approach could ultimately help us to design and validate novel therapeutics for the betterment of MS patients.

## Author Contributions

PD, AR, and AW wrote the manuscript. AA edited the manuscript. MR wrote and edited the manuscript.

## Conflict of Interest Statement

MR holds an EMD Serono Canada and endMS Research and Training Network Transitional Career Development Award from the MS Society of Canada and the Multiple Sclerosis Scientific Research Foundation. Acceptance of this unrestricted educational grant by the MS Society of Canada does not constitute endorsement by researcher or society of any product(s) of EMD Serono Canada. Funds from this award did not support the work contained herein.

## Abbreviations

B6, C57BL6/J; CNS, central nervous system; CSF, cerebrospinal fluid; EAE, experimental autoimmune encephalomyelitis; HLA, human leukocyte antigen; MBP, myelin oligodendrocyte glycoprotein; MOG, myelin oligodendrocyte glycoprotein; MS, multiple sclerosis; NOD, non-obese diabetic; PLP, proteolipid protein; RR, relapsing-remitting; SCID, severe combined immunodeficient; SP, secondary progressive; T1D, type 1 diabetes; TcR, T cell receptor; Tg, transgenic; Th, T helper; WT, wild-type.
